# Estimation of Left Ventricular Ejection Fraction Using Cardiovascular Hemodynamic Parameters and Pulse Morphological Characteristics with Machine Learning Algorithms

**DOI:** 10.3390/nu14194051

**Published:** 2022-09-29

**Authors:** Shing-Hong Liu, Zhi-Kai Yang, Kuo-Li Pan, Xin Zhu, Wenxi Chen

**Affiliations:** 1 Department of Computer Science and Information Engineering, Chaoyang University of Technology, Taichung City 41349, Taiwan; 2 Division of Cardiology, Department of Internal Medicine, Chang Gung Memorial Hospital, Chiayi Branch, Chiayi City 61363, Taiwan; 3 College of Medicine, Chang Gung University, Taoyuan City 33305, Taiwan; 4 Heart Failure Center, Chang Gung Memorial Hospital, Chiayi Branch, Chiayi City 61363, Taiwan; 5 Division of Information Systems, School of Computer Science and Engineering, University of Aizu, Aizu-Wakamatsu City, Fukushima 965-8580, Japan

**Keywords:** heart failure, left ventricular ejection fraction, cardiovascular hemodynamic parameter, morphological characteristic of pulse, machine learning

## Abstract

It is estimated that 360,000 patients have suffered from heart failure (HF) in Taiwan, mostly those over the age of 65 years, who need long-term medication and daily healthcare to reduce the risk of mortality. The left ventricular ejection fraction (LVEF) is an important index to diagnose the HF. The goal of this study is to estimate the LVEF using the cardiovascular hemodynamic parameters, morphological characteristics of pulse, and bodily information with two machine learning algorithms. Twenty patients with HF who have been treated for at least six to nine months participated in this study. The self-constructing neural fuzzy inference network (SoNFIN) and XGBoost regression models were used to estimate their LVEF. A total of 193 training samples and 118 test samples were obtained. The recursive feature elimination algorithm is used to choose the optimal parameter set. The results show that the estimating root-mean-square errors (E_RMS_) of SoNFIN and XGBoost are 6.9 ± 2.3% and 6.4 ± 2.4%, by comparing with echocardiography as the ground truth, respectively. The benefit of this study is that the LVEF could be measured by the non-medical image method conveniently. Thus, the proposed method may arrive at an application level for clinical practice in the future.

## 1. Introduction

The body relies on the pumping action of the heart to deliver blood with rich oxygen and nutrients to the cells to maintain its functions. When the heart cannot supply enough blood to the cells, the body will feel weak and short of breath. Then, people will have difficulty performing some daily activities such as climbing stairs, carrying groceries, and even walking [1]. Heart failure (HF) means that the heart does not pump properly. Most patients with HF are associated with abnormal heart contraction and relaxation because their hearts have myocardium hypertrophy and fibrosis. The diagnostic methods for HF usually use the cardiac biomarker (B-type natriuretic peptide, BNP), and the performance of heart contraction indicated by the left ventricular ejection fraction (LVEF) [2].

LVEF is defined as the ratio of stroke volume (SV) to end diastolic volume (EDV) of the left ventricular, which is a measurement of change in the contractility under conditions of constant load [3]. In the clinical practices for LVEF measurement, the medical image methods include two or three-dimensional echocardiography, nuclear imaging, cardiac computed tomography, and cardiac magnetic resonance imaging [4]. The two-dimensional echocardiography is the most popular method among them. All of these methods are expensive and available only in medical settings. If the HF patients are not carefully treated, their mortality approaches 50% within five years. How to measure the heart contractility every day conveniently at home will be a challenging topic and beneficial for patients with HF.

The cardiovascular circulative system could be described by the Windkessel model that shows the relation between the blood pressure (BP), cardiac output (CO), and systemic vascular resistance (SVR) [5,6,7]. The BP, SV and CO are fundamental measures of cardiovascular functions, and are essential for accurate understanding of cardiovascular pathophysiology, and the guidance of fluid mechanics [8]. Liu et al. used the pulse contour of the brachial artery based on the Windkessel model to estimate the SV values for 55 subjects and compared to the echocardiography. The results showed a high correlation coefficient of *r* = 0.693 [9]. Liu et al. also used this method to measure the changes of SV before and after the passive leg raising test for 24 subjects and compared to the impedance cardiography. The results showed a higher correlation coefficient of *r* = 0.842 [10]. Moreover, the pulse contour analysis (PCA) includes the time and pressure parameters of the heart’s pumping action [11,12,13,14], which could be used to evaluate the characteristics of the cardiovascular system, such as blood pressure, blood flow, left ventricular ejection time, vascular stiffness, etc. However, some studies showed that the CO measured by PCA could not be recommended to assess the CO values of HF patients whose heart has a different load and EDV conditions [15,16].

Machine learning (ML) algorithms have been widely used in physiological measurements for estimating the physiological parameters, such as blood pressure [11,17], muscle mass [18], calories [19,20], glucose [21], stroke volume [22], classifying the signal qualities of electrocardiogram [23] and photoplethysmogram [24,25], detecting arrythmia [26] and risky activities in daily life [27]. When using an ML method to process the regression or classification problem, searching the major features and finding the appropriate ML algorithms will depend on the collected data [28,29]. The feature processing is an important issue, which can directly affect the performance of the ML algorithm. The more accurate the features, the higher performance of the ML algorithm. Although some traditional statistical analysis methods have good results for clinical prediction in some cases, ML methods reignite the interest in exploiting these fields [30,31].

HF patients in the treatment not only need the drug to control their blood pressure, relax the walls of blood vessels, and reduce the heart rate [32], but also have to change their life style in prevention and management of hypertension, which include the sodium restriction, alcohol restriction, body weight reduction, smoking cessation, proper diet, and exercise adoption [33]. Thus, they need an apparatus to monitor their heart function every day. However, the blood pressure monitor is the only apparatus for the HF patients currently. In this study, we propose a novel machine learning-based method to estimate LVEF using the physiological parameters including cardiovascular, morphological, and bodily information.

## 2. Materials and Methods

The goal of this study is to use the ML method for estimating the LVEF of HF patients with the cardiovascular hemodynamic parameters, morphological characteristics of pulse and bodily information. There were twenty patients who participated in this study. They all had chronic HF disease, and had been treated for many years. The LVEF measured by two-dimension echocardiography was used as the ground truth to evaluate the performance of the proposed method. A special blood pressure monitor not only measured the hemodynamic parameters, but also recorded eight seconds of the blood pressure signal [10]. Thirty-three parameters were acquired. We used the optimal feature selection algorithm to search the important parameters as the input features to two ML algorithms, XGboost [34] and self-constructing neural fuzzy inference network (SoNFIN) [35], to estimate the LVEF.

**Figure 1:**
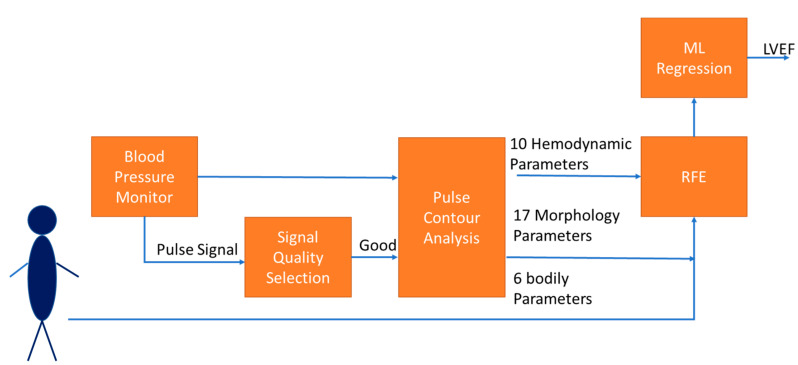
The framework of estimating LVEF in this study includes collecting 33 parameters, extracting optimal features by RFE, and estimating LVEF by ML regression.

Figure 1 shows the framework in this study. A blood pressure monitor could measure ten hemodynamic parameters and record the blood pressure signal [9,10]. A decision rule for the signal quality was designed to select the pulse waves with good quality. The PCA was used to extract ten hemodynamic parameters and seventeen morphological parameters from the high-quality pulses [24]. Six parameters of bodily information were included. The optimal parameters were determined by the recursive feature elimination (RFE). Finally, two ML models used these parameters to estimate the LVEF.

### 2.1. Cardiovascular Hemodynamic Parameters

**Figure 2:**
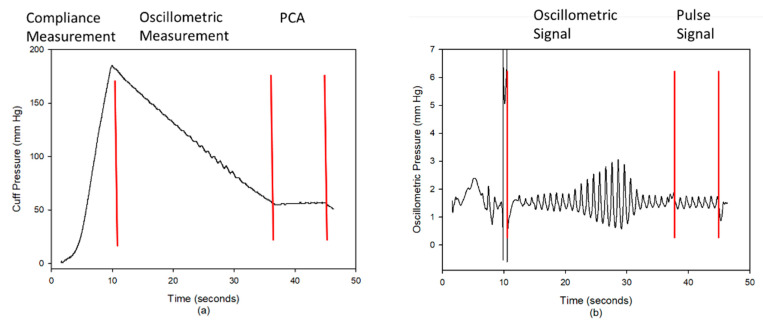
(**a**) The cuff pressure has three phases, inflating duration (compliance measurement), deflating duration (oscillometric measurement), and holding duration (pulse contour analysis, PCA). (**b**) The oscillometric signal was used to measure the blood pressure, and the pulse signal was used to measure the SV.

Liu et al. proposed a pulse contour method to measure the cardiac hemodynamic parameters, which was implemented in a blood pressure monitor (iBP-130, Biostart, Taiwan) [9,10]. This apparatus has two sensors measuring the cuff pressure and pumping air flow. The digital pressure and flow sensors are FPS 520 and FDF 400 (Formosa Measurement Technology Inc. Ltd., Taipei city, Taiwan). The pressure signal is filtered by two infinite impulse response filters with the different bandwidth for the oscillometric blood pressure measurement and pulse contour analysis. The bandwidths of the filters are 0.3 Hz to 4 Hz for the blood pressure measurement, and 0.3 Hz to 20 Hz for PCA. The sampling rate was 125 Hz. Figure 2 shows the measurement procedure of this apparatus, including the building of the cuff model [36], oscillometric measurement [37], and PCA [9]. The signal of the cuff pressure is shown in Figure 2a, and its filtered signal is shown in Figure 2b. In the inflating duration (about 10 s), the compliance (C) of the brachial artery is measured. In the deflating duration, the heart rate (HR), systolic blood pressure (SBP), diastolic blood pressure (DBP), pulse pressure (PP) and mean artery pressure (MAP) are measured by the oscillometric method (about 25 s). In the duration of PAC (about 8 s), the SV is measured. The CO is obtained by multiplying HR and SV. These hemodynamic parameters are also normalized by the body surface area (BSA), including the stroke volume index (SI), and cardiac output index (CI). Thus, ten hemodynamic parameters are totally acquired.

### 2.2. Morphological Parameters of Pulse

In the duration of PCA, the pulse wave is easily coupled with the artificial motion when the cuff pressure is held at about 55 mmHg. The pulse quality would affect the accuracy of physiological measurement [24,38,39]. Thus, we proposed a decision rule to evaluate the quality of each pulse wave in the duration of PCA. Then, the morphological parameters of the pulse with a good quality were extracted.

#### 2.2.1. Pulse Quality Analysis

**Figure 3:**
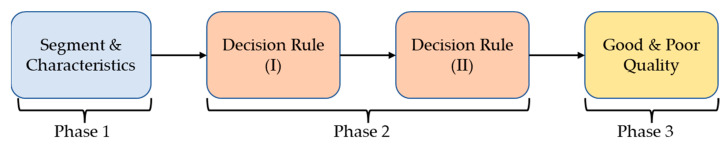
The flowchart of pulse quality analysis. Phase 1 is the pulse segmentation and the four characteristics determination. Phase 2 is to apply the decision rules for evaluating the pulse quality. Phase 3 is to mark the quality of each pulse.

**Figure 4:**
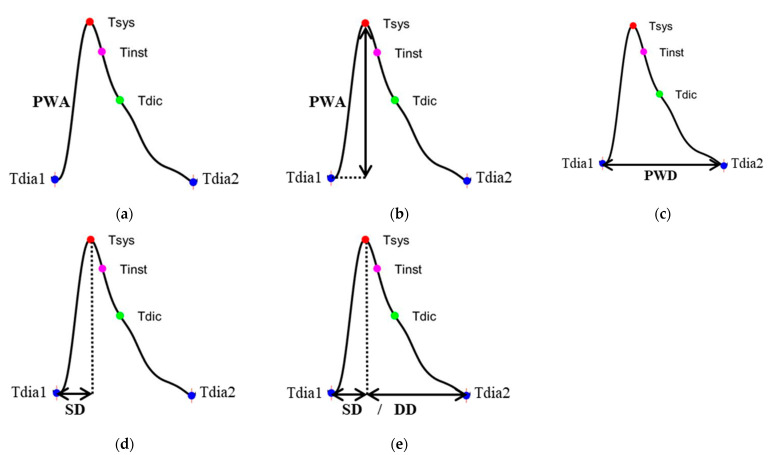
(**a**) The four characteristics of pulse, (**b**) the pulse wave amplitude (PWA), (**c**) the pulse wave duration (PWD), (**d**) the systolic duration (SD) of pulse wave, and (**e**) the ratio of SD and diastolic duration (DD).

**Figure 5:**
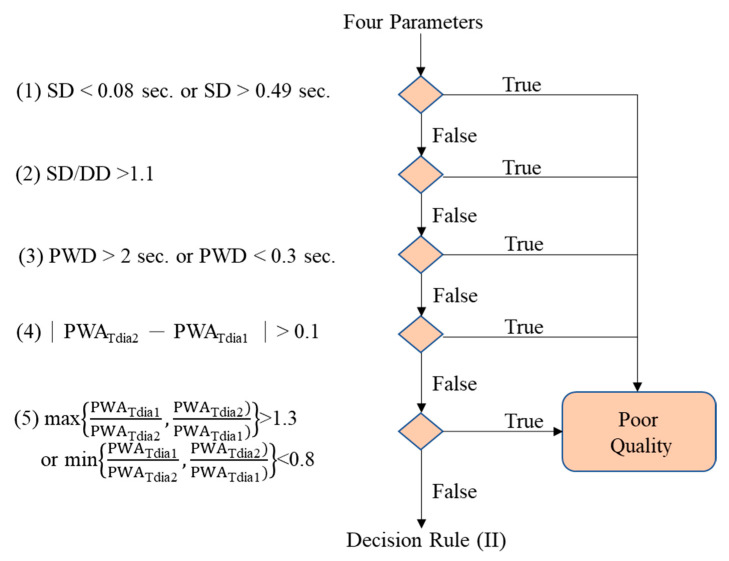
The decision rule (I). If any one rule is true, the pulse is poor quality.

**Figure 6:**
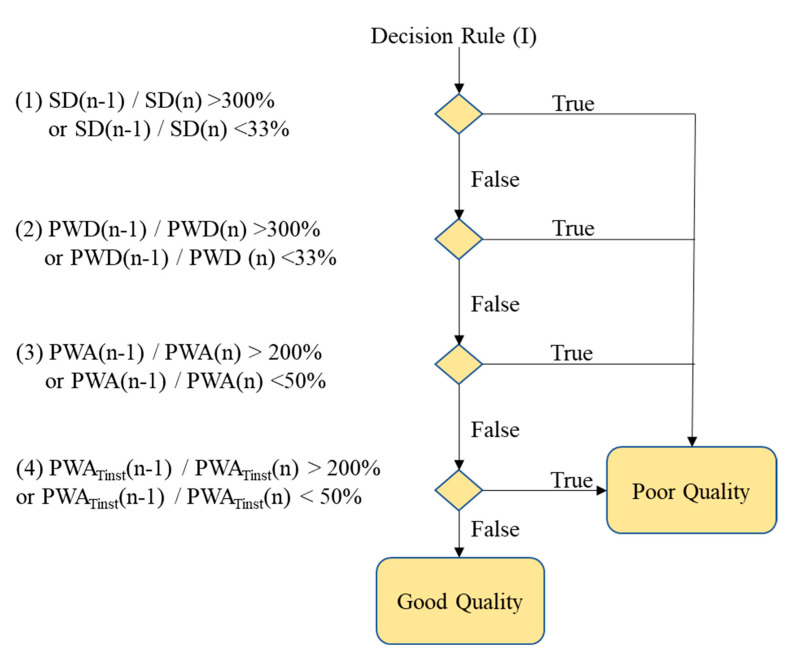
The decision rule (II). If any one rule is true, the pulse is poor quality.

**Figure 7:**
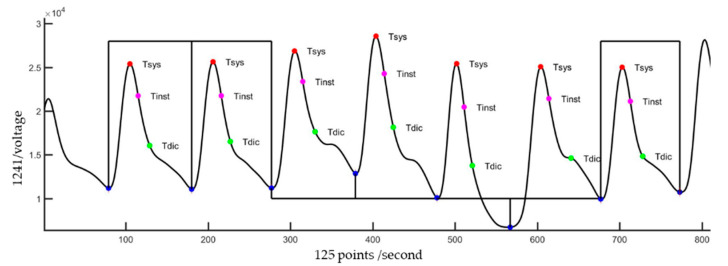
A signal segment in the duration of PCA has seven pulses. When the quality of the pulse wave is good, a high level is marked in the corresponding cycle. Otherwise, a low level is marked.

Figure 3 shows the flowchart of the pulse quality analysis. In the first phase, each pulse wave is segmented and four characteristic points are determined, including main peak (Tsys), foot (Tdia), dicrotic notch (Tdic), and systolic ending time (Tinst), as shown in Figure 4a [26,40]. Four parameters, pulse wave amplitude (PWA, Figure 4b), pulse wave duration (PWD, Figure 4c), systolic duration (SD, Figure 4d), and ratio of systolic and diastolic durations (SD/DD, Figure 4e), are defined. In the second phase, two decision rules are used to determine the quality of each pulse by the four parameters. Figure 5 shows the flowchart of decision rule (I) based on the four parameters. If one rule is true, the quality of this pulse wave is poor. Figure 6 shows the flowchart of decision rule (II) that finds the change of three parameters of neighbor pulses. *n* represents the current pulse, and *n-1* represents the previous pulse. If one rule is true, the quality of this pulse wave is poor. In the third phase, the quality of each pulse is defined. Figure 7 shows a pulse signal that includes seven heart beats. When the baseline is wandering, the four pulses are marked as the poor qualities (low level). The other three pulses are marked as the good qualities (high level). Only the pulses with good qualities were used to detect the SV and morphological parameters. The same types of pulse parameters were averaged as the values of this measurement. 

#### 2.2.2. Morphological Parameters

**Figure 8:**
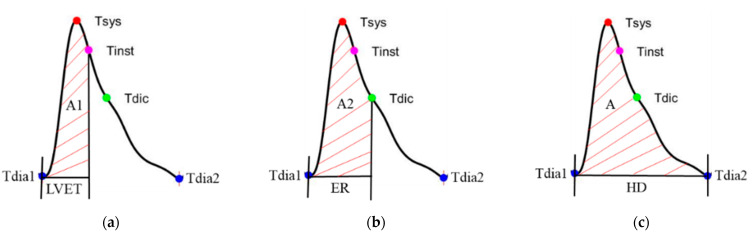
(**a**) The left ventricular ejection time (LVET) is defined at the systolic ending time (Tinst), the integral area of which is A1, (**b**) the ejection relaxation time (ER) is defined at the dicrotic notch time (Tdic), the integral area of which is A2, and (**c**) the total area is defined as A.

The pulse wave was calibrated by the blood pressure as the blood pressure wave. According to the four characteristics, we defined three different integral areas of the pressure wave under the three different durations, as shown in Figure 8. The left ventricular ejection time (LVET) is defined at the systolic ending time (Tinst), the integral area of which is A1, as shown in Figure 8a. The ejection relaxation time (ER) is defined at the dicrotic notch time (Tdic), the integral area of which is A2, as shown in Figure 8b. The total area is defined as A3, as shown in Figure 8c. 

When the heart contracts, the volume of the left ventricle has an absolute relationship with these area and time-related parameters [12]. In order to improve the predictive performance of the models, we extended these parameters through ratios. Table 1 shows the four different ratios, time to time, time to area, area to time, and area to area. There are ten parameters. Moreover, Romano’s method proposed a pressure wave profile as changes of pressure with time along each cardiac cycle [12], *P*/*t*,

(1) $$P/t=\frac{P_{sys}-P_{dia2}}{T_{sys}}+\frac{P_{dic}}{T_{dia2}-T_{dic}}-\frac{P_{inst}}{T_{dia2}-T_{inst}}$$

Thus, the total number of morphological parameters is 17.

### 2.3. Bodily Information

The BSA has a high relation with the total body water [41], which is usually used to normalize the CO and SV for reducing the individual difference [42]. Moreover, body mass index (BMI) describes a normal range of the relation between weight and height. A higher BMI could reduce the recovery of LVEF for the HF patients [43,44]. In this study, six bodily parameters, including gender, age, height, weight, BMI and BSA, were used.

### 2.4. Features Extraction and Regression

In total, 33 parameters were used to estimate the LVEF by two supervised regression approaches, XGboost and SoNFIN. In order to reduce redundancy of the features. The RFE was used to search the optimal parameter set as the input feature to estimate the LVEF [18,45].

#### 2.4.1. Features Extraction

All training samples were used to evaluate the optimal parameters. In order to reduce the flag problems like overfitting or selection bias, the RFE uses the five-fold cross validation. The RFE fitted the XGboost model that did not perform the adjustment of optimal parameters to remove the weakest parameters until reaching the specified number of parameters. All features were ranked by root-mean-square error (E_RMS_), and by recursively eliminating a parameter with the lowest E_RMS_ per loop. The lower the impact feature, the lower the change of E_RMS_. Thus, RFE could eliminate the parameters with the dependencies and collinearity existing in the model. Table 2 shows the E_RMS_ under the different number of parameters for the lowest three E_RMS_. We find that the nine parameters, SBP, CI, CO, C, A1/A, DBP, ER/HD, MAP, BSA, has the lowest E_RMS_. Thus, these parameters are the feature to search the optimal parameter of XGBoost and estimate the LVEF.

#### 2.4.2. XGBoost

XGBoost is a gradient boosting tree model that integrates many tree models to form a strong classification and regression tree (CART) [46]. The CART assumes that the tree is a binary tree and divides the features continuously. For example, the current tree node is split based on the *i*-th input variable *x_i_*, and the samples with the variable less than *s* are divided into the left subtree (*R*_1_), and the samples larger than *s* are divided into the right subtree (*R*_2_),

(2) $$R_{1}\left(i,s\right)=\left\{x|x_{i}\leq s\right\}\;\mathrm{and}\;R_{2}\left(i,s\right)=\left\{x|\;x_{i}>s\right\}.$$

The CART essentially divides the sample space in the feature dimension, and the optimization of this space division is a NP-complete problem. The objective function generated by a typical CART is,

(3) $$\sum_{xi\in R_{m}}(y_{i}-f{\left(x_{i}\right)}^{2}),\;$$

where *f* is a nonlinear function, *y_i_* is the *i*-th target output. Therefore, we solve the best divisive feature *i* and the best divisive point *s* by minimizing the objective function,

(4) $$\underset{j,s}{\min}[\underset{c_{1}}{\min}\sum_{x_{i}\in R_{1}\left(j,s\right)}{\left(y_{i}-c_{1}\right)}^{2}+\underset{c_{2}}{\min}\sum_{x_{i}\in R_{2}\left(j,s\right)}{\left(y_{i}-c_{2}\right)}^{2}],$$

where *C*_1_ and *C*_2_ are the results of the branch. The theorem of XGBoost is to continuously add trees and continuously perform feature splitting to grow a tree. Each time a tree is added, it is actually learning a new function to fit the residual of the last prediction. When we obtain *N* trees after training, we need to predict the score of a sample. In fact, according to the characteristics of this sample, each tree will fall to a corresponding leaf node. One leaf node corresponds to a score. The total scores corresponding to all trees represent the predicted value of the sample.

The grid-search method was used to find the optimal parameters of XGBoost. Table 3 shows the range of each parameter and its step. The final results were that the learning rate is 0.07, maximum depth is 3, minimum child weight is 5, gamma is 0.2, subsample is 1, subsample ratio is 1, reg_alpha is 0, and reg_lambda is 0.

#### 2.4.3. Self-Constructing Neural Fuzzy Inference Network

SoNFIN is a 5-layer fuzzy neural network. The fuzzy model of SoNFIN can be represented by the following expression [25]:

$$\mathrm{Rule}\;j:\;\mathrm{If}\;x_{1}\;is\;A_{1j}\;\mathrm{and}\cdots \mathrm{and}\;x_{n}\;is\;A_{nj}$$

$$\mathrm{Then},\;y_{j}\;\mathrm{is}\;w_{0j}+\sum_{i=1}^{n}w_{ij}x_{i}$$

where *A_ij_* is a fuzzy set, and $w_{0j}+\sum_{i=1}^{n}w_{ij}x_{i}$ is the traditional Takagi–Sugeno–Kang model. The five layers are described in detail as follows.

Layer 1: Each node in this layer corresponds to one parameter of feature. Thus, the number of input nodes is nine. The input feature is transmitted forward to the next layer directly:

(5) $$u_{i}^{\left(1\right)}=x_{i}$$

Layer 2: For the fuzzy set *A_ij_*, a Gaussian membership function is used to describe the degree that the input variable *x_j_* belongs to the *i*-th fuzzy set. Its mathematical function is defined as follows:

(6) $$u_{ij}^{\left(2\right)}=\exp(\frac{-{\left[u_{i}^{\left(1\right)}-m_{ij}\right]}^{2}}{\sigma_{ij}^{2}})$$

where *m_ij_* and *σ_ij_* are the center and width of the membership function, respectively. This function is implemented by each node.

Layer 3: A node in this layer represents one fuzzy logic rule and performs precondition matching of a rule. We employ the multiplication in each Layer 3 node:

(7) $$u_{j}^{\left(3\right)}=\prod_{i}u_{ij}^{\left(2\right)}$$

Layer 4: Nodes in this layer are called the consequent nodes. The linear association of weights in this layer is as follows:

(8) $$u_{j}^{\left(4\right)}=u_{j}^{\left(3\right)}(w_{0j}+\sum_{i=1}^{n}w_{ij}x_{i})$$

Layer 5: Each node in this layer corresponds to one output variable. The node integrates all the actions recommended by Layer 5 and acts as a defuzzifier by the equation below:

(9) $$u_{j}^{\left(4\right)}=u_{j}^{\left(3\right)}(w_{0j}+\sum_{i=1}^{n}w_{ij}x_{i})$$

In the training phase, SoNFIN performs the structure training and parameter training, concurrently. Initially, there were no rules in the SoNFIN. For the structure training, a default value, H, was used as a criterion for the generation of fuzzy rules. When the output of Layer 3 was below to H for every rule, a new rule was generated. Therefore, more rules were generated for a smaller value of H. The initial width of each Gaussian fuzzy set was assigned to a default value, σ. To train the parameters, the objective is to minimize the error function (*V_error_*),

(10) $$V_{error}={\left(y-o\right)}^{2}$$

where *y* is the target output. The consequent part and the fuzzy-set parameters were tuned by a recursive least-squares method and a gradient-descent method, respectively. The details of the training algorithm were found elsewhere [35]. In the study, the default *H* and σ were set to 10^−15^ and 0.008. The learning rate was set to 0.005.

### 2.5. Statistical Analysis

The root-mean-square error (E_RMS_) and coefficient of determination (R^2^) were used to evaluate the performance of this study. E_RMS_ is an index to find the difference between the estimated value and target value, which is described below,

(11) $$E_{RMS}=\sqrt{\frac{1}{n}\sum_{i=1}^{n}{\left(y_{i}-{\hat{y}}_{i}\right)}^{2}}$$

where *n* is sample number, *y* is the target value, and $\hat{y}$ is the estimated value. The coefficient of determination in statistics represents the proportion of the variance in the dependent variable predicted from the independent variable, which indicates the level of variation in the given data set.

(12) $$R^{2}=1-\frac{\sum_{i}{\left(y_{i}-\bar{y}\right)}^{2}}{\sum_{i}{\left(y_{i}-\hat{y_{i}}\right)}^{2}}$$

where $\bar{y}$ is the mean of all samples.

### 2.6. Data Collection

In this study, there were twenty patients (male: 16, female: 4) with the symptoms of heart failure who had been measured the LVEF by the 2D echocardiography (Philips IE33, Philips Healthcare, Netherlands, US) at least three times. The interval between two LVEF measurements was at least one month apart. In general, these patients were hospitalized, whose blood pressures were measured by the iBP-130 blood pressure monitor, concurrently. These data were used as the training samples. Moreover, they also only measured the blood pressure some other days. These data were used as the testing samples. Their age was between 39 and 84 years (66.8 ± 13.7 years, mean ± standard deviation), body weight (BW) was between 41 and 98 Kg (62.4 ± 11.8 Kg), body height (BH) was between 154 and 174 cm (163.4 ± 5.8 cm), SBP was between 135 and 79 mmHg (110.8 ± 11.8 mmHg), and DBP was between 38 and 83 mmHg (68.9 ± 10.2 mmHg). Table 4 shows the basic characteristics of 20 patients. The data collection protocol was approved by the Research Ethics Committee of Chang Gung Medical Foundation Institutional Review Board (No. 201701357B0C602), Taipei, Taiwan.

The number of training samples was 193 sets. The number of testing samples was 118 sets. In the training samples, the LVEF measured by echocardiography was the target output. However, in the testing samples, because patients did not measure the LVEF by the echocardiography, there were not real target outputs. We hypothesized that the change of LVEF was slow within one year. Therefore, during two inpatient treatments, the testing target outputs were estimated by linear interpolation of the training target outputs.

## 3. Results

The training model used the five-fold cross validation to evaluate the performances. The model with the best result was used to estimate LVEF. In testing results, we estimated the LVEF values of each patient within six or nine months. The Bland–Altman plots were used to compare the performance of SoNFIN and XGBoost models.

### 3.1. Training Models

**Figure 9:**
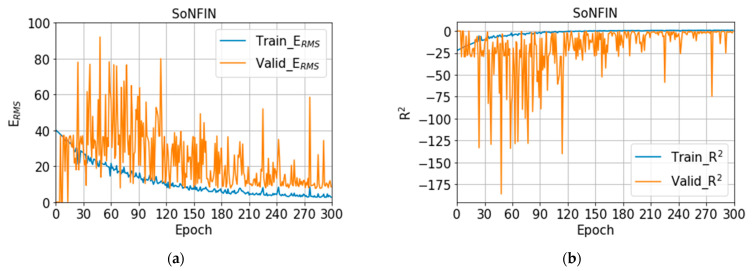
(**a**) The training (blue) and validation (orange) curves of E_RMS_, (**b**) the training (blue) and validation (orange) curves of R^2^ with SoNFIN.

**Figure 10:**
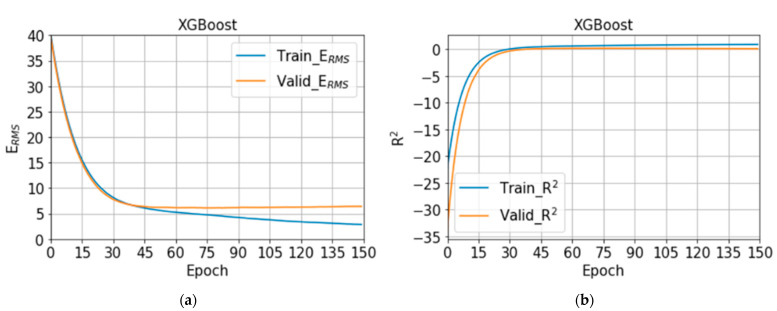
(**a**) The training (blue) and validation (orange) curves of E_RMS_, (**b**) the training (blue) and validation (orange) curves of R^2^ with XGBoost.

For SoNFIN, E_RMS_ is 12.79 ± 4.07%, and R^2^ is −1.77 ± 1.83. The training (blue) and validation (orange) curves of E_RMS_ and R^2^ are shown in Figure 9a,b, separately. When the number of epochs is 125, E_RMS_ and R^2^ for validation have the lowest value, 7.61% and −0.28. However, we find that the E_RMS_ and R^2^ approach to a stable status when the number of epochs is 300. Therefore, we chose the model at 300 epochs. For XGBoost, E_RMS_ is 17.94 ± 0.99%, and R^2^ is 0.02 ± 0.11. The training (blue) and validation (orange) curves of E_RMS_ and R^2^ are shown in Figure 10a,b, separately. When the number of epochs is 74, E_RMS_ and R^2^ for validation have the lowest value, 6.11% and 0.18. However, we find that the E_RMS_ and R^2^ approach to a stable status when the number of epochs is 100. Therefore, we chose the model at 100 epochs.

### 3.2. Testing Models

**Figure 11:**
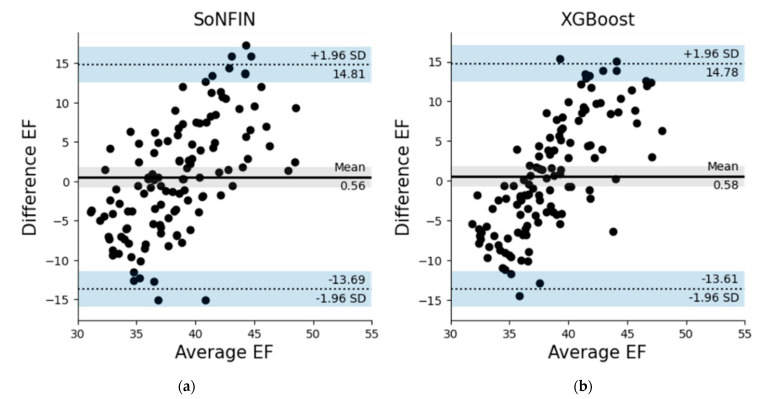
Bland–Altman plots for (**a**) SoNFIN, and (**b**) XGBoost.

Table 5 shows the estimated LVEF values of 20 patients by the SoNFIN and XGBoost within three intervals. The numbers of testing samples in the three intervals are 55, 33 and 30 sets, respectively. Six patients have two intervals only. The E_RMS_ of SoNFIN and XGBoost are 6.9 ± 2.3% and 6.4 ± 2.4%. For SoNFIN, the E_RMS_ of patient 5 has the smallest value, 3.15%, and patient 9 has the largest value, 10.10%. For XGBoost, the E_RMS_ of patient 1 has the smallest value, 2.05%, and patient 10 has the largest value, 11.13%. Bland–Altman plots for SoNFIN and XGBoost are shown in Figure 11. The mean and standard deviation (mean ± sd) of the differences were 0.56 ± 7.27% and 0.58 ± 7.24% for SoNFIN and XGBoost, respectively. We find that the means of two models are close, and all data are within the limits of agreement, although there are five data for SoNFIN and three data for XGBoost fall outside of the limitations, as shown in Figure 11.

## 4. Discussion

In this study, we used the hemodynamic parameters, morphological parameters of pulse and bodily information to estimate the LVEF with the machine learning algorithms. In Table 2, the nine parameters, SBP, CI, CO, C, A1/A, DBP, ER/HD, MAP, and BSA, have the best performance. In these parameters, six parameters belong to the cardiovascular hemodynamics, two parameters are the characteristics of pulse contour, and the body information has one. The area (A1) under the systolic duration of the blood pressure wave is proportional to SV, and the total area (A) is proportional to EDV [47]. Moreover, the ED is the time of heart ejection, and HD is the time of heart beat. The A1/A and ED/HD parameters are considered proportional to the ratio of SV and EDV under the pressure and time scales. The pressure parameters including the SBP, DBP, and MAP have the high relation with LVEF [48]. The SBP lower than 120 mmHg is associated with reduced cardiac ejection fraction (HFrEF) in coronary arteries. In this study, the statistical analysis for the SBP and DBP of patients were 110.8 ± 11.8 mmHg and 68.9 ± 10.2 mmHg, respectively. Thus, the blood pressure parameters were the important feature to estimate the LVEF. Moreover, the CO and CI represent the function of heart blood flow. The lower CI, the lower LVEF [33]. We found that the compliance (C) of peripheral artery was also an important parameter for estimation of LVEF. An increase in inflammatory markers is found in HF patients, which is a condition characterized by chronic low-level inflammation, and would sustainably affect the cardiovascular function [49,50]. The patients in this study were the chronic HF, so the compliances of their peripheral arteries would be stiff. BSA is a more accurate indicator of a metabolic mass that is estimated as a fat-free mass [51]. Thus, BSA usually is used as the normalization of hemodynamic parameters. Thus, the nine parameters are in line with the LVEF pathophysiology.

The reduced LVEF is a good characteristic of HF, which is also an index for the effective therapies for HF patients [52]. In ESC HF guidelines in 2016, the mid-range LVEF (HFmrEF) of HF is defined as LVEF 40–49% [53]. Then, according the ranges of LVEF, there are four HFrEF categories, LVEF < 20%, 20–25%, 26–34% and 35–39%. Thus, a categorical range of HFrEF is decreasing by about 5% to 10%. The E_RMS_ values of SoNFIN and XGBoost for the LVEF estimation were 6.9 ± 2.3% and 6.4 ± 2.4%, which just were on the range boundary. We thought that three reasons could be discussed. Firstly, the target outputs of testing data were not measured by the echocardiography, which also were estimated by the interpolation method between two LVEF values by the echocardiography. Secondly, the patients in this study not only had the chronic HP, but also had the other chronic diseases, like as diabetes, kidney disease, or atherosclerosis, etc. These diseases would affect the changes of the nine parameters. Thus, the estimated model would have a better performance if the model is made by the personal data. Thirdly, the number of samples is too few. The numbers of training and testing samples were only 193 and 118. If there are more samples, the performance of our proposed method will be better.

**Figure 12:**
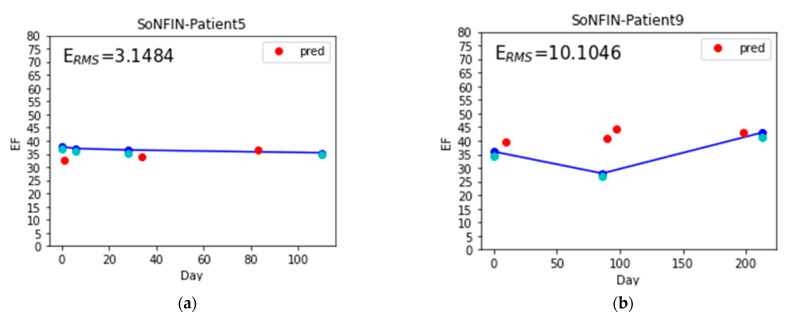
The estimated LVEF for the lowest and highest E_RMS_ values by SoNFIN, the blue points are the LVEF measured by the echocardiography, green points are the estimated LVEF of training model, and red points are the estimated LVEF of the testing model, (**a**) patient 5, and (**b**) patient 9.

**Figure 13:**
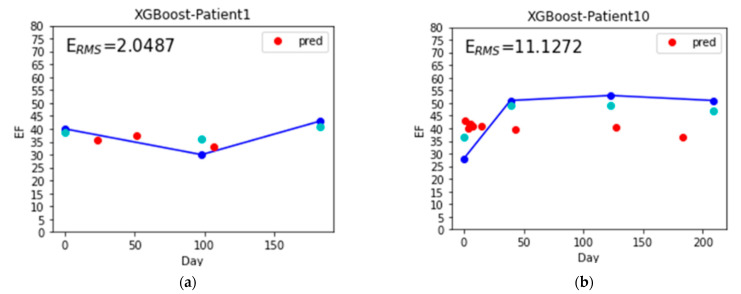
The estimated LVEF for the lowest and highest E_RMS_ values by XGBoost, the blue points are the LVEF measured by the echocardiography, green points are the estimated LVEF of training model, and red points are the estimated LVEF of the testing model, (**a**) patient 1, and (**b**) patient 10.

The coefficients of determination (R^2^) for the SoNFIN and XGBoost were −0.77 ± 1.83 and 0.02 ± 0.11, which is close to 0. This meaning is the estimated value closing to the average value of samples. We examined the all data, and found that variability of LVEF is low because the patients had the chronic HF and were treated for a long time. Their heart functions were controlled well by the drug and diet. Figure 12 shows the estimated LVEF for the lowest (patient 5, Figure 12a) and highest (patient 9, Figure 12b) E_RMS_ values by SoNFIN. The blue points are the LVEF measured by the echocardiography, green points are the estimated LVEF of training model, and red points are the estimated LVEF of the testing model. The variability of LVEF for patient 9 is larger than patient 5. Figure 13 shows the predicted LVEF for the lowest (patient 1, Figure 13a) and highest (patient 10, Figure 13b) E_RMS_ values by XGBoost. The variability of LVEF for patient 10 is larger than patient 1.

As LVEF is assumed to be a measure of myocardial contractility for the long-standing, it could be used to evaluate the heart function of HF patients [3]. However, the widespread classification of patients with HF is based on whether LVEF is preserved (HFpEF) or reduced (HFrEF). For the HFpEF, patients have the HF signs and symptoms, but LVEF would be larger than 45% or 50% [52]. In this study, the participated patients all belonged to HFrEF, whose LVEF values were not larger than 50%. Thus, the first limitation was the proposed method only for patients with reduced HF. Moreover, we did not recruit healthy subjects without the signs and symptoms of HF and whose LVEF values were greater than 50%. Therefore, we could not distinguish the measurement deviation between healthy and unhealthy groups. That is the second limitation for this study.

## 5. Conclusions

The LVEF is an important index to evaluate the heart function of HF patients, and is usually measured by the medical image method. This study proposed a cheaper method using the cardiovascular hemodynamic parameters, morphological parameters of pulse, and bodily information to estimate LVEF with the machine learning algorithms. Based on the RFE, the optimal nine parameters, SBP, CI, CO, C, A1/A, DBP, ER/HD, MAP, and BSA, were explored, which all conform the LVEF pathophysiology. Although the E_RMS_ of estimated LVEF was satisfactory enough, the number of samples does not support the performance of our method arriving to an application level for clinical practice. In the future, we will collect more data to improve our method.

## Figures and Tables

**Table 1:** The ten extended parameters including four different ratios, time to time, time to area, area to time, and area to area.

Ratio	Parameter	Ratio	Parameter
Time to Time	LVET/HD	Area to Area	A1/A
	ER/HD		A2/A
Time to Area	LVET/A1	Area to Time	A1/LVET
	ER/A2		A2/ER
	HD/A		A/HD

**Table 2:** The lowest three E_RMS_ under the different number of parameters.

Number	Parameter	E_RMS_ (%)
9	SBP, CI, CO, C, A1/A, DBP, ER/HD, MAP, BSA	5.80
	LVET, Pt, SBP, SV, C, HR, ER/HD, MAP, BSA	6.53
	SBP, SV, CI, CO, C, HR, ER/HD, MAP, BSA	6.59
8	Pt, SBP, A2/A, HR, HD, ER/HD, MAP, BSA	6.34
	Pt, SBP, SV, C, HR, ER/HD, MAP, BSA	6.42
	SBP, SV, C, HR, DBP, ER/HD, MAP, BSA	6.45
7	SBP, SV, C, DBP, ER/HD, MAP, BSA	6.43
	SBP, SV, CI, C, ER/HD, MAP, BSA	6.54
	Pt, SBP, SV, HR, ER/HD, MAP, BSA	6.70
6	SBP, CI, C, R/HD, MAP, BSA	6.42
	SBP, C, DBP, ER/HD, MAP, BSA	6.48
	SBP, C, HR, ER/HD, MAP, BSA	6.51
5	SBP, SV, HR, HD, MAP	6.51
	Pt, SBP, SV, HR, MAP	6.51
	SBP, C, ER/HD, MAP, BSA	6.54

**Table 3:** In the grid-search method, the ranges of each XGBoost parameter and their steps.

Parameters	Range	Step	Final Value
Learning rate	(0.01, 0.2)	0.01	0.07
Maximum depth	(2, 5)	1	3
Minimum child weight	(1, 10)	1	5
gamma	(0.0, 1.0)	0.1	0.2
subsample	(0.0, 1.0)	0.1	1
subsample ratio	(0.0, 1.0]	0.1	1
reg_alpha	(0.0, 1.0)	0.1	0
reg_lambda	(0.0, 1.0)	0.1	0

**Table 4:** The basic characteristics of twenty patients.

Patient	Gender	Age (Years)	BH (cm)	BW (kg)	SBP (mmHg)	DBP (mmHg)
1	M	39	174	72	79	38
2	M	77	164	54	109	61
3	M	62	166	66	116	67
4	M	68	165	98	109	82
5	M	64	160	77	106	83
6	M	78	168	67	135	76
7	M	79	168	59	99	61
8	M	48	175	73	99	67
9	F	84	156	53	121	63
10	M	67	166	61	93	64
11	M	79	167	59	133	72
12	M	82	158	56	115	73
13	M	80	162	59	127	64
14	F	54	155	41	116	73
15	M	79	159	63	115	72
16	M	69	168	66	107	69
17	M	67	161	63	107	70
18	M	66	154	54	103	62
19	F	49	162	51	111	79
20	F	44	160	57	117	82

**Table 5:** The E_RMS_ of estimated LVEF for 20 patients by SoNFIN and XGBoost within three intervals.

Patient	Interval I (*N*)	Interval II (*N*)	Interval III (*N*)	SoNFIN	XGBoost
				E_RMS_ (%)	
**1**	**2**	**1**		6.60	**2.05**
2	0	3	1	4.12	6.09
3	3	3		5.07	6.06
4	7	1	2	4.24	3.93
5	1	0	2	**3.15**	2.5
6	3	1	2	6.63	6.88
7	1	1	2	9.21	10.58
8	3	0	2	8.36	6.71
9	1	3		**10.10**	7.07
10	6	1	2	9.44	**11.13**
11	0	2	5	4.81	4.65
12	3	2		7.40	7.57
13	3	1	2	8.85	8.2
14	5	2	2	7.04	6.95
15	3	1	2	9.38	8.94
16	2	0	2	10.08	6.33
17	4	1	2	6.41	7.13
18	2	5	2	8.88	9.67
19	4	3		3.74	4.9
20	2	2		4.56	5.37
Sum mean ± sd	55	33	30	6.9 ± 2.3	6.4 ± 2.4

Note: *N* indicates number of samples.
